# Prediction of *in vivo* prenatal chlorpyrifos exposure leading to developmental neurotoxicity in humans based on *in vitro* toxicity data by quantitative *in vitro–in vivo* extrapolation

**DOI:** 10.3389/fphar.2023.1136174

**Published:** 2023-03-07

**Authors:** Engi Abdelhady Algharably, Emma Di Consiglio, Emanuela Testai, Francesca Pistollato, Anna Bal-Price, Abdulkarim Najjar, Reinhold Kreutz, Ursula Gundert-Remy

**Affiliations:** ^1^ Institute of Clinical Pharmacology and Toxicology, Charité—Universitätsmedizin Berlin, Corporate Member of Freie Universität Berlin and Humboldt-Universität zu Berlin, Berlin, Germany; ^2^ Mechanisms, Biomarkers and Models Unit, Environment and Health Department, Istituto Superiore di Sanità, Rome, Italy; ^3^ European Commission, Joint Research Center (JRC), Ispra, Italy; ^4^ Beiersdorf AG, Hamburg, Germany

**Keywords:** PBK modelling, reverse dosimetry, animal alternative, dose-response modeling, organophosphorus pesticides, new approach methodologies (NAMs)

## Abstract

**Introduction**: Epidemiological studies in children suggested that in utero exposure to chlorpyrifos (CPF), an organophosphate insecticide, may cause developmental neurotoxicity (DNT). We applied quantitative *in vitro*–*in vivo* extrapolation (QIVIVE) based on *in vitro* concentration and non-choline esterase-dependent effects data combined with Benchmark dose (BMD) modelling to predict oral maternal CPF exposure during pregnancy leading to fetal brain effect concentration. By comparing the results with data from epidemiological studies, we evaluated the contribution of the *in vitro* endpoints to the mode of action (MoA) for CPF-induced DNT.

**Methods:** A maternal-fetal PBK model built in PK-Sim^®^ was used to perform QIVIVE predicting CPF concentrations in a pregnant women population at 15 weeks of gestation from cell lysate concentrations obtained in human induced pluripotent stem cell-derived neural stem cells undergoing differentiation towards neurons and glia exposed to CPF for 14 days. The *in vitro* concentration and effect data were used to perform BMD modelling.

**Results:** The upper BMD was converted into maternal doses which ranged from 3.21 to 271 mg/kg bw/day. Maternal CPF blood levels from epidemiological studies reporting DNT findings in their children were used to estimate oral CPF exposure during pregnancy using the PBK model. It ranged from 0.11 to 140 μg/kg bw/day.

**Discussion:** The effective daily intake doses predicted from the in vitro model were several orders of magnitude higher than exposures estimated from epidemiological studies to induce developmental non-cholinergic neurotoxic responses, which were captured by the analyzed *in vitro* test battery. These were also higher than the *in vivo* LOEC for cholinergic effects. Therefore, the quantitative predictive value of the investigated non-choline esterase-dependent effects, although possibly relevant for other chemicals, may not adequately represent potential key events in the MoA for CPF-associated DNT.

## 1 Introduction

The development and application of physiologically based kinetic (PBK) models have gained momentum in recent years in biomedical research as well as in chemical risk assessment providing insight into the dosimetry related to observed health risks in humans (Paini et al., 2019). The current paradigm shift in chemical risk assessment from animal testing to next-generation risk assessment (NGRA) aims at integrating novel, human-relevant, toxicity pathway-based alternative new approach methodologies (NAMs) including *in silico* methods, among which PBK modelling (Zhang et al., 2018). Though promising, the data generated from *in vitro* toxicity approach by using alternative cell or organoid-based of human origin systems could only be made useful with the application of quantitative in vitro-to-in vivo extrapolation (QIVIVE) (Zhang et al., 2018). PBK modelling provides a framework to help the translation of effect concentrations eliciting biological responses in a human-relevant *in vitro* system to *in vivo* exposure levels (Yoon et al., 2012). A combined approach of QIVIVE application utilizing PBK and benchmark dose (BMD) modelling could be used to identify a concentration value (then considered as point of departure-PoD) corresponding to exposure levels in human tissue associated with perturbations of toxicity pathways *in vitro* (Louisse et al., 2017; Zhang et al., 2018). Central to a successful QIVIVE is the selection of a human-relevant endpoint in an *in vitro* assay conducted with a robust study design (Rogiers et al., 2020). This will help define a reliable reference point from *in vitro* concentration-response data before extrapolating it to an *in vivo* apical endpoint alteration (Rogiers et al., 2020). In cases in which the toxicological profile of a chemical is not known, selecting an adequate endpoint in an *in vitro* test system can, however, become quite challenging. PBK modelling is also a valuable tool to evaluate internal exposures of chemicals in situations where information on the kinetics is sparse or lacking particularly in special populations, e.g., pregnant women, where kinetic changes affect drug/chemical disposition determining both maternal and fetal exposure (Coppola et al., 2021).

Chlorpyrifos (CPF), an organophosphorus pesticide, can readily cross the placenta and affect fetal growth and neurodevelopment (Abdel-Rahman et al., 2002; Saulsbury et al., 2008) with effects such as altered cognition, motor control, and behavior in rats and mice (Burke et al., 2017; EFSA, 2019). As evidenced by epidemiological studies, the *in utero* exposure to CPF, has been linked to developmental neurotoxicity (DNT) with effects appearing in children later in life such as learning disabilities, attention deficit/hyperactivity disorders, decrease in intelligent quotient and working memory (Rauh et al., 2006; Rauh et al., 2011; Burke et al., 2017). A pregnancy-adapted PBK model including the fetus can help predict CPF levels reaching the developing fetal brain and its possible accumulation over long-term exposure.

In this work, we aimed to examine the feasibility and predictivity of *in vitro* toxicity testing data applying a QIVIVE approach. We set to predict maternal doses, leading to the *in vitro* effect concentration in the target tissues, i.e., fetal brain, by performing PBK modelling-based reverse dosimetry. Applying a combined QIVIVE-dose-response approach for some selected *in vitro* toxicodynamic non-cholinergic endpoints described in (Di Consiglio et al., 2020) (i.e., neurite outgrowth, synaptogenesis, BDNF levels, percentages of neurons and astrocytes), we obtained the corresponding maternal doses. They were compared to doses modelled by using the maternal CPF blood concentrations associated with neurodevelopmental adverse outcomes in children from epidemiological studies. The result of the comparison allowed us to assess the contribution of the *in vitro* toxicodynamic effects other than the well-established mechanism of acetylcholinesterase (AChE) inhibition to the CPF mode of action (MoA) for DNT. By this, we could examine the potential of using QIVIVE and validate the predictive value of *in vitro* models for CPF-induced DNT.

## 2 Materials and methods

### 2.1 Acquirement of *in vitro* concentration–response data in a human induced pluripotent stem cell-derived neuronal/glial model.

#### 2.1.1 Kinetics data

We used published data on CPF from an *in vitro* biokinetic and toxicodynamic study (Di Consiglio et al., 2020) in a cellular model suitable to model some critical events of human brain development. In this study, human induced pluripotent stem cell (hiPSC)-derived neural stem cells (NSCs) undergoing differentiation toward neurons and astrocytes were repeatedly treated twice a week for 14 days with CPF at a nominal concentration of 21 μM, corresponding to IC_5_ (a nominal concentration causing 5% reduction of viability after 14 days). The cellular processes such as neuronal/glial differentiation, neurite outgrowth and synaptogenesis shown by the *in vitro* model of differentiating cells correspond to the neurodevelopmental processes seen approximately by the 13th postconceptual week (Silbereis et al., 2016). The biokinetic profile of CPF and its toxic metabolite CPF-oxon (CPFO) in all the *in vitro* compartments (medium, cells and plastic device) was followed for 24 h on the first (day 1) and the last time CPF treatment and medium were refreshed (i.e., day 11) prior to terminating the experiment after a total of 14 days. Along the 24 h, four time points were selected (1, 3, 6, and 24 h) to measure the actual concentration of the parent compound (CPF) and the metabolite CPFO in the medium and in the cell lysate (Di Consiglio et al., 2020). For our modelling purposes, the measured concentrations in the medium were considered to represent the plasma/blood concentration and the concentrations in the cell lysate were considered to represent the concentration in the target organ, i.e., the fetal brain. To perform the PBK modelling, the *in vitro* concentrations, which were expressed as concentrations per well (nmol/well) in the publication (Di Consiglio et al., 2020), were recalculated as concentrations in μg/mL.

### 2.1.2 Dynamics data

In the same study, a number of toxicodynamic biomarkers were measured to assess DNT effects induced by CPF after 14 days repeated exposure at the nominal concentrations of 18.45, 21.21, 24.39, 28.05, 32.26, and 37.10 μM (Di Consiglio et al., 2020). These included measurements for neurite outgrowth (neurite length, number of neurites/neuron and number of branch points/neurite), synapse formation, BDNF (brain derived neurotrophic factor) levels, spontaneous electrical activity generation assessed by multielectrode array (MEA) analysis, and analysis of neuronal and astrocyte cell percentages by high content imaging of *β*-III-Tubulin and glial fibrillary acidic protein (GFAP) staining, respectively, in order to evaluate the overall neuronal cell development and function.

### 2.2 Human maternal-fetal PBK model

We used the Open Systems Pharmacology (OSP) Suite to build a model for a pregnant woman and her fetus. We started with a reference PBK model for CPF for an adult non-pregnant woman in PK-Sim^®^ incorporating the standard model structure comprising 18 compartments (Krauss et al., 2012). The compartments represent organs/tissues, and the different organs were connected by the arterial blood flow and venous blood flow. Physicochemical properties of CPF (molecular weight, 350.57 g/mol; logP_O/W_, 4.96; solubility, 1.4 mg/L) retrieved from PubChem and a non-protein-bound fraction of 0.03 (Timchalk et al., 2002) were implemented in the molecule building unit (Table 1). For parametrizing absorption, we used a specific intestinal permeability for CPF taken from an *in vitro* study that assessed the intestinal uptake of CPF using single-pass intestinal perfusion method in rats (Cook and Shenoy, 2003). This was later optimized by fitting the model to the observed *in vivo* data where a 10-fold lower value improved the predictions of the model. Tissue partition coefficient was modelled according to Schmidt algorithm (Schmitt, 2008a; Schmitt, 2008b) implemented in the software (Table 1; Supplementary Table S1). CPF input was modelled by the oral route as single dose exposure whereas elimination was only through the hepatic route. CPF is converted by CYP450-mediated biotransformation through oxidative desulfuration and dearylation primarily into CPFO and 3,5,6-trichloro-2-pyridinol (TCPy) metabolites, respectively (Tang et al., 2001). Metabolism was assumed to occur *via* CYP2B6 to give the CPFO (being the most active for desulfuration pathway) and *via* CYP2C19 to give TCPy (being the most active for desulfuration pathway) and is described using Michaelis-Menten kinetics. The corresponding metabolic parameters, Vmax and Km for the two pathways were taken from an *in vitro* study in hepatic human microsomes (Zhao et al., 2019) (Table 1). Other CYP isoforms have been reported to contribute to the oxidative desulfuration and dearylation of CPF to fom the oxon and TCPy *in vitro*, respectively, including CYP1A2 as well as other isoforms such as 2A6, 2C9, 2D6, and 3A4 (Buratti et al., 2002; Buratti et al., 2003). However, the contribution of these isoforms, particularly, 2A6, 2C9, 2D6, and 3A4, to the overall formation of these metabolites may be regarded as not significant (Eaton et al., 2008), hence, we did not account for them in the PBK model.

**TABLE 1:** Summary of physicochemical and physiological input parameters for CPF-PBK model.

Relevant physicochemical parameters	Value
Molecular weight (g/mol)	350.6^a^
Log*P*o:w	4.96^b^
p*K*_a_	Non-dissociable
Solubility (mg/mL)	0.0014^a^
Relevant kinetic input parameters	
Absorption	P_eff_ 6.16 e^−5^ cm/sec^c^ *f*_a_ 0.32 (Calculated *in silico*)
Distribution	
Fu	0.03^d^
Tissue/blood partition coefficient	Calculated *in silico* ^e^
Clearance (hepatic)^f^	
Metabolic constants	
CYP 2B6 mediated-desulfuration	14.04 (nmol/min/mg)^g^
*K*_*m*_	4.33 (µM)^g^
*V*_*max*_	9.36 (nmol/min/mg)^g^
CYP 2C19 mediated-dearylation	28.5 (µM)^g^
*K*_*m*_	
*V*_*max*_	

CYP, cytochrome P450; K_m,_ Michaelis–Menten constant; pKa, acid dissociation constant; P_eff_, specific intestinal permeability; *f*_*a*_, fraction absorbed; fu, fraction unbound.

^a^ PubChem (accessed March 2021).

^b^ Gebremariam et al. (Gebremariam et al., 2012).

^c^ Cook et al. (Cook and Shenoy, 2003).

^d^ Timchalk et al., 2002.

^e^ According to (Schmitt, 2008a; Schmitt, 2008b).

^f^ Intrinsic clearance scaled *in silico*.

^g^ Zhao et al. (Zhao et al., 2019).

The predictive performance of the model was evaluated by comparing the results of simulations with observed plasma concentration of CPF *in vivo* in human volunteers applying oral doses 1–2 mg/kg bw (Brzak, 2000; Timchalk et al., 2002).

**FIGURE 1:**
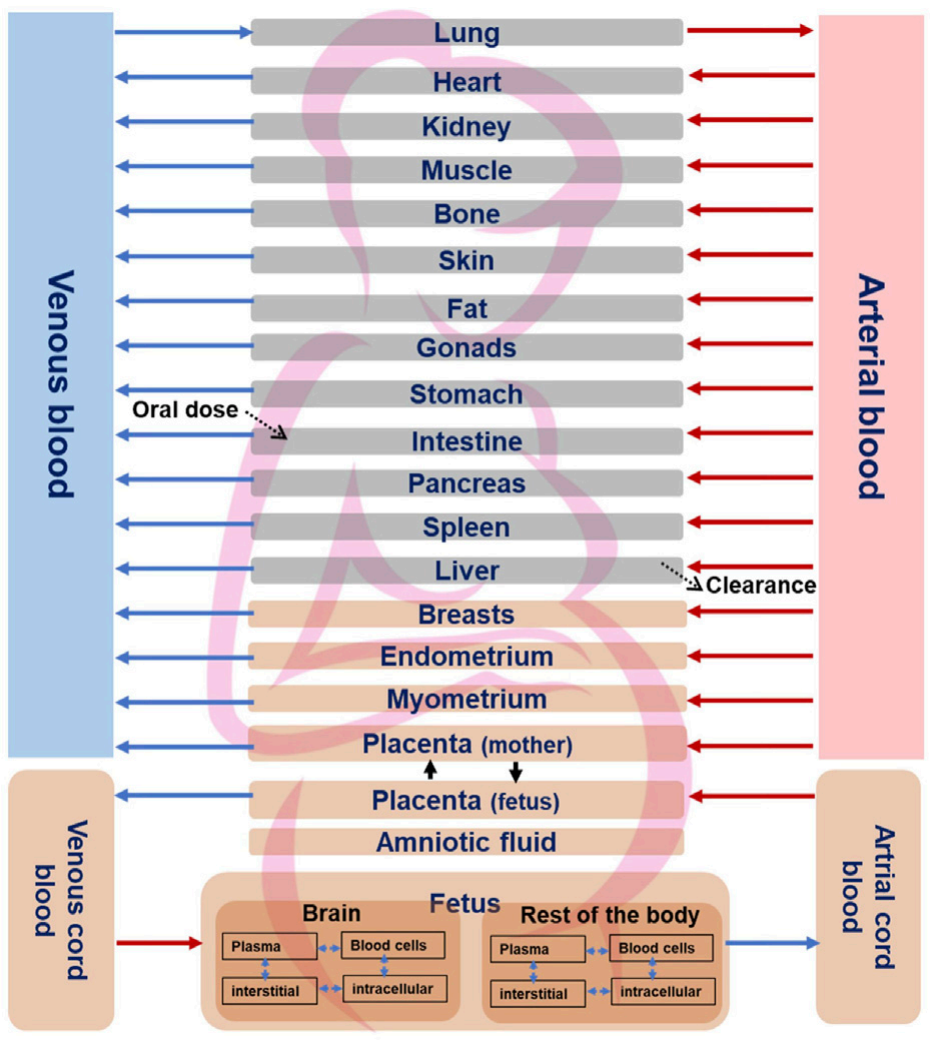
Structure of the fetal-maternal PBK model for CPF. Pregnancy-specific compartments are depicted in orange. The four sub-compartments (blood cells, plasma, interstitial, and intracellular) for CPF distribution are shown for brain and rest of the fetal body sub-compartments. Solid lines indicate blood flow process, dotted lines indicate transport *via* passive diffusion.

Once the non-pregnant PBK model was established and its predictivity considered satisfying, all drug-specific parameters were kept, and pregnancy-specific changes were incorporated into the model which included nine pregnancy-specific compartments. Furthermore, we extended the model to include relevant fetal sub-compartments, i.e., fetal brain (the target tissue) and a lumped fetal compartment (rest of the body) in MoBi^®^ as previously described (Dallmann et al., 2018b) (Figure 1). Fetal metabolism of CPF mediated by CYP3A isoforms have been demonstrated *in vitro* (Buratti et al., 2006). This applies particularly to CYP3A7 isoform which is expressed during the fetal life and was shown to have catalytic activity towards CPF bioactivation into CPFO *in vitro* (Buratti et al., 2006). However, owing to the early stage of pregnancy (end of the first trimester), and the immaturity of fetal liver, fetal hepatic metabolism of CPF could be ignored. Similarly, pregnancy-related changes in the expression and activity of the involved CYP-enzymes were regarded as minimal as data indicate prominent changes only in later stages of pregnancy (Pariente et al., 2016). Therefore, starting values were kept as those in the non-pregnant model. The adapted pregnant PBK model was exported to PK-Sim^®^ for carrying out population simulations in pregnant women at the respective gestational age. The physiological parameters, i.e., organ weights and blood flows for the pregnancy-specific organs corresponding to the same stage of the *in vitro* model cell differentiation regarding the human brain development, i.e., 13th postconceptual week or 15th week of gestation, were retrieved from the literature and implemented in the model (Dallmann et al., 2018a). We parameterized the organ weight and blood flow for the fetal brain sub-compartment using data from a previous study (Archie et al., 2006). Blood flow and organ weight for the “rest of the body” subcompartment were derived by subtracting the fetal brain data for blood flow and organ weight from the total blood flow and weight of the fetus organ, respectivley, which were taken from the literature (Abduljalil et al., 2021). Of note, due to unavailability of CPF kinetics data in pregnant women, predictive performance of pregnant PBK model could not be tested. However, the differences of the modelled concentration-time profiles of pregnant-women (at 15 weeks of gestation) and non-pregnant-women were evaluated based on the knowledge of the changing physiology in pregnancy. Thereafter, simulation for CPF time-concentration course in both the mother’s plasma and the fetal brain were performed in a pregnant population (N = 100) at 15 weeks of gestation.

### 2.3 Prediction of *in vivo* dose–response using the PBK model-facilitated reverse dosimetry approach

The biokinetic data from the *in vitro* study were used to calculate the average measured daily concentration, i.e., C_average_, for CPF as the relevant dose metric. In this respect, the area under the concentration-time curve was calculated and then divided by the time period over which the concentration points were taken, e.g., for CPF nominal concentration 21 μM, which was used as a placeholder for the calculation of the C_average_ for the rest of the nominal concentrations used in the toxicodynamic part of the *in vitro* study. Applying reverse dosimetry, the oral maternal dose at steady state was predicted which would lead to the same intracellular C_average_ in the developing fetal brain after repeated dose administration over 1 month to reach steady state as *in vitro* repeated dosing has been performed. In the next steps, we converted the *in vitro* nominal concentrations used in the toxicodynamics experiment of Di Consiglio et al. (Di Consiglio et al., 2020) into actual concentrations using the relationship as established in the kinetic part of the same publication. These were then used to calculate the corresponding *in vivo* doses for the human fetus. By performing this exercise, *in vitro* concentration–response data were translated into *in vivo* dose–response data for the DNT endpoints.

### 2.4 Benchmark dose (BMD) analysis of predicted *in vivo* dose–response data

The generated *in vivo* dose–response data were used to perform a BMD analysis using the R-package PROAST (version 70.1) (www.proastweb.rivm.nl) to obtain lower and upper Benchmark doses (BMDL and BMDU), indicating minimal and low effect doses, respectively. Models for continuous data were used and a benchmark response (BMR) of an effect size of one standard deviation (SD) of the background response for each assessed neurological endpoint was applied (EFSA et al., 2017; Slob, 2017). The 90% confidence interval around the BMD was estimated with a lower bound (BMDL) and upper bound (BMDU). To check for the uncertainty that may be further introduced by conducting the QIVIVE on the PBK-predicted oral doses, we applied benchmark analysis directly on the concentration-response curve obtained from the *in vitro* study relating nominal concentrations and the toxicodynamic endpoints to estimate the upper limit of benchmark concentration (BMCU). This was converted later by reverse dosimetry into BMDU The goodness of fit application of the models was used to determine if the model could be accepted with *p* > 0.05. All models which met the requirements for acceptance of the model fit were considered by model averaging to derive a single BMD confidence interval from the set of BMD confidence intervals for each neurotoxicity endpoint (Supplementary Figure S1). The BMDU was selected to predict a dose able to cause adverse effects on the brain which will be compared with exposures doses associated with DNT from epidemiological studies. In these studies, maternal/cord blood levels of CPF were reported which were the target of the prediction of maternal CPF exposure doses during pregnancy. Using the kinetic model, we also simulated the concentrations in the fetus corresponding to the concentrations measured in the mothers in these studies.

### 2.5 Relevance of the *in vitro* model for quantitative prediction of DNT

The predictive value of the *in vitro* DNT model was evaluated by comparing the exposure dose of CPF obtained for pregnant women (i.e., BMDU) with those reported in epidemiological studies to be required to induce developmental neurotoxic responses. Eligible studies were those including neurodevelopmental effects measured in the offspring of pregnant women and CPF blood concentration from the mothers (Rauh et al., 2006; Silver et al., 2017; Chiu et al., 2021). This allowed assessing the suitability of the selected *in vitro* experimental system and the endpoints tested to predict CPF-induced DNT in quantitative terms.

## 3 Results

### 3.1 Kinetic model evaluation

**FIGURE 2:**
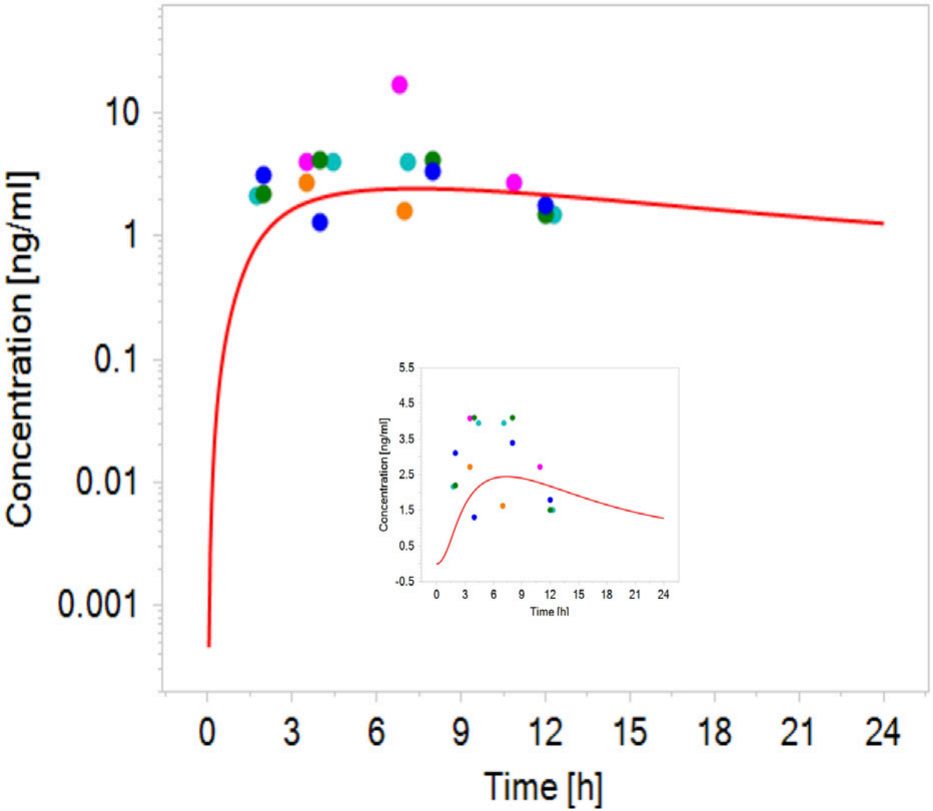
Simulated (line) concentration–time profile of CPF following oral administration of 2 mg/kg bw compared with the observed plasma concentration–time data in volunteers administered oral CPF at a dose level of 2 mg/kg bw (points) (Brzak, 2000; Timchalk et al., 2002).

**FIGURE 3:**
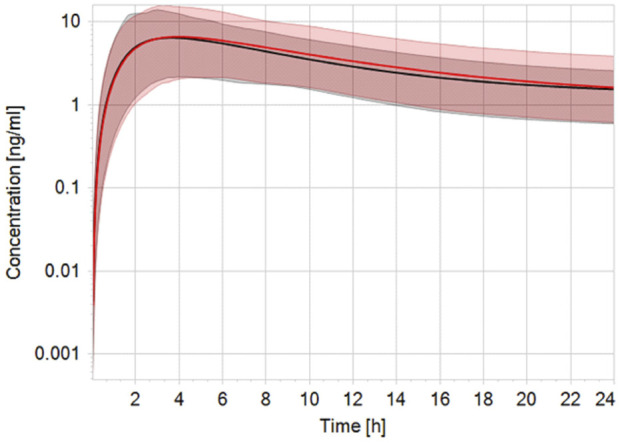
Simulated plasma CPF concentration-time profile in non-pregnant (black line) vs. pregnant (red line) population (*n* = 100) after a single oral dose of 2 mg/kg bw. The black and red solid lines represent predicted mean profile while the shaded areas represent the predicted 95% confidence interval.

Robust human data on the kinetics of CPF are scarce. In a controlled study by the US EPA (Brzak, 2000), CPF was given orally to male and female volunteers in doses 1–2 mg/kg bw. CPF concentrations in blood could be quantified only in some of the subjects. In those subjects, the concentrations ranged from 1.1 to 5.6 ng/g and 1.3–18 ng/g in the 1.0 and 2.0 mg/kg bw group, respectively. Similarly, Timchalk et al. (Timchalk et al., 2002) studied on a small scale the kinetic profile of CPF in male and female volunteers following oral CPF administration at dose levels of 1 and 2 mg/kg bw. Our PBK model predicted plasma concentration-time profiles of CPF with 1 and 2 mg/kg bw oral doses in adult human females and males and were in good agreement with concentration-time profiles observed in the human studies (Brzak, 2000; Timchalk et al., 2002). Figure 2 shows the model performance comparing simulated plasma concentration with the observed data for the 2 mg/kg bw dose level. The performance of the developed CPF model in non-pregnant women was considered to be in agreement with the observed data; however, the evaluation of the pregnant model was not feasible since no kinetic studies performed on pregnant women exposed to CPF were reported in the literature. Nevertheless, only small differences were observed between concentration-time profiles of pregnant and non-pregnant women, which are explained by the expected physiological changes (Costantine, 2014) (Figure 3).

### 3.2 PBK modelling-reverse dosimetry based on C_average_ from the lysate of human NSCs undergoing differentiation towards neurons/glia

The observed *in vitro* average CPF intracellular concentration after 14-day repeated exposure to 21 μM nominal concentration was 7942.3 ng/mL and the corresponding oral maternal dose calculated by QIVIVE was 20.5 mg/kg bw/day. The *in vitro* nominal concentration of 21 µM CPF (corresponding to an IC_5_) was considered as the lowest-observed-adverse-effect concentration (LOAEC) specific for the analysis of synaptogenesis after repeated treatment in the *in vitro* study (Di Consiglio et al., 2020). Maternal oral doses leading to the *in vitro* intracellular concentrations were thus predicted and a dose-response relationship for each toxicodynamic endpoint was established (Table 2).

**TABLE 2:** *In vitro* actual cellular average CPF concentrations in cultured human NSCs undergoing differentiation towards neurons and glia, corresponding to the nominal dosing levels (data taken from (Di Consiglio et al., 2020) and *in vivo* oral maternal doses obtained by reverse dosimetry.

In vitro nominal concentration (µM)	Actual Caverage (ng/mL)	Dose resulting from reverse dosimetry (mg/kg bw/day)
18.45	6,977.6	8.9
21	7942	20.5
21.21	8021.4	22
24.39	9224	65.5
28.05	10,608	239
32.26	12,200	1,080
37.1	14,031	5,000

NSCs, neural stem cells.

The tested nominal concentrations were between 18.45 and 37.1 µM. After converting them into actual concentrations and using the pregnant model for QIVIVE, doses were obtained which we used for BMD modelling with a BMR derived from the SD of the respective measurement. The predicted maternal BMDUs for the *in vitro* toxicodynamic effects ranged from 3.21 to 271 mg/kg bw/day (Table 3).

**TABLE 3:** BMD confidence interval and total maternal oral dose predicted based on average CPF concentration as kinetic metric for the investigated toxicodynamic markers.

Toxicodynamic marker	BMDlower (mg/kg bw/day)	BMDupper (mg/kg bw/day)
**Neurite/neuron**	17	271
**Branch points**	2.79	81.6
**BDNF levels**	0.283	10.7
**Synapses**	0.695	12.8
**% B-III-Tubulin + cells**	0.121	7.52
**% GFAP + cells**	0.197	3.21

BMD, benchmark dose; BDNF, brain derived neurotrophic factor; GFAP, glial fibrillary acidic protein.

**FIGURE. 4:**
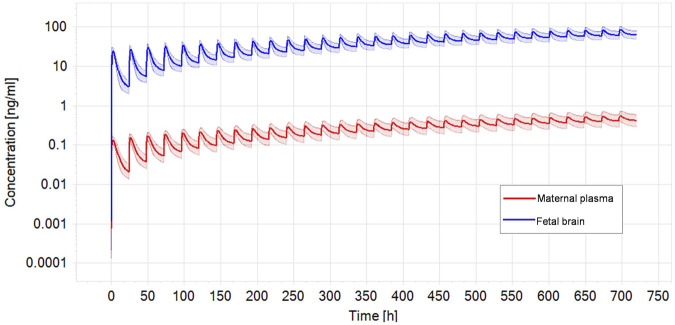
Optimized dose trial in reverse dosimetry for 0.56 ng/mL CPF plasma concentration in the mother according to Silver et al. (Silver et al., 2017) after 1 month repeated oral exposure in a population of pregnant women (N = 100). Solid line represents simulated mean CPF profile while shaded areas denotes the predicted 95% confidence interval.

Performing benchmark analysis on the nominal concentration-response curve, BMCU ranged from 15 to 27.4 µM which corresponded to oral daily doses of 7.2–233 mg/kg bw/day, respectively, using PBK-reverse dosimetry (Supplementary Table S2). The values obtained with the two approached were very similar, excluding possible additional uncertainty introduced by the reverse dosimetry-BMD modelling approach. CPF blood concentration in exposed pregnant women ranged from 0.13 ng/mL to 7.33 ng/mL in the publications we could find (Rauh et al., 2006; Rauh et al., 2011; Silver et al., 2017; Chiu et al., 2021) (Table 4). In the epidemiological studies, maternal exposures were not reported. Therefore, we performed reverse dosimetry in the pregnant women population using the PBK model to calculate maternal doses leading to the blood concentrations in the mothers reported in those studies. The predicted oral doses were 11, 2.8, 0.2, and 0.11 μg/kg/day corresponding to the blood levels of 0.56 ng/mL, 0.13 ng/mL, 10.6 pg/g and 5 pg/g measured in the studies, respectively (Rauh et al., 2006; Rauh et al., 2011; Silver et al., 2017; Chiu et al., 2021). Figure 4 shows one reverse dosimetry trial for 0.56 ng/mL reported by Silver et al. (Silver et al., 2017). Furthermore, we also simulated fetal brain concentrations resulting from the obtained maternal doses (Table 4). The corresponding simulated concentrations (reported as the arithmetic mean) in the fetal brain were 85.97–1,147.6 (Silver et al., 2017), 21.40 (Chiu et al., 2021), 1.75 (Rauh et al., 2006) and 0.85 ng/mL (Rauh et al., 2011) (Table 4). A difference of several orders of magnitude can be observed by comparing the BMDU for any of the toxicodynamic biomarkers the *in vitro* study associated with subtle neurodevelopmental adverse effects with the doses showing neurodevelopmental effects obtained by reverse dosimetry from epidemiological studies. Similarly, the simulated fetal brain concentrations were much lower than any of the *in vitro* average concentrations triggering the toxicodynamic endpoint.

**TABLE 4:** PBK model-predicted oral maternal doses and CPF concentrations in fetal brain corresponding to reported CPF levels in epidemiological studies.

Epidemiological study with neurodevelopmental effects in infants	CPF blood concentration in exposed pregnant women	Maternal oral dose by RD (mg/kg bw/day)	Simulated concentration in the fetal brain (ng/mL)	BMDU for DNT endpoints (mg/kg bw/day)
**Silver et al. (2017)**	0.56–7.33 (ng/mL)	0.011–0.14	85.97–1,147.6	3.21–271
**Chiu et al. (2021)**	0.13 (ng/mL)	0.0028	21.40	
**Rauh et al. (2006)**	10.6 pg/g	0.0002	1.75	
**Rauh et al. (2011)**	5 pg/g	0.00011	0.85	

RD, reverse dosimetry; BMDU, upper limit of benchmark dose; DNT, developmental neurotoxicity.

## 4 Discussion

*In vitro* and *in silico* approaches which facilitate the understanding of toxicodynamic processes are increasingly applied to move away from traditional animal testing (Coppola et al., 2021). The use of *in silico* methods such as PBK modelling serves as a predictive tool for any substance systemic exposure and could be valuable to support risk assessment when data are sparse, such as in pregnant women (Coppola et al., 2021). In this work, we performed QIVIVE to evaluate the quantitative predictive value of an vitro test system for DNT effects which was assumed to be promising in this respect (Pistollato et al., 2020; Pistollato et al., 2021; Nunes et al., 2022), using non-acetyl cholinesterase-dependent markers in hiPSC-derived neuronal/glial cell model.

The PBK model was successful in predicting the *in vivo* concentration-time profile for CPF for non-pregnant women, validating the kinetic model and the substance specific and physiological parameters (in non-pregnant women). Parameterizing a PBK model can become challenging when *in vivo* kinetic data are limited or totally lacking to calibrate and validate model predictions as was the case with CPF in pregnant women.

The human test system applied in the *in vitro* study (Di Consiglio et al., 2020) mimics key neurodevelopmental processes including neural cell proliferation, neuronal/glial differentiation, neurite outgrowth and early synaptogenesis, which can be observed in the first trimester in humans (Tau and Peterson, 2010). Due to the early stage of pregnancy in which the model was set (at the end of the first trimester), pregnancy-related anatomical and physiological changes, e.g., metabolizing enzymes, were not yet fully expressed and less likely to impact CPF kinetics. This was evidenced by the fact that the predicted concentration-time profile of CPF in pregnant women was only slightly different from that in non-pregnant women and the slight differences were in accordance with the expected differences due to pregnant-related changes in physiology at the indicated gestational age.

Using the maternal-fetal PBK population model, we modeled the *in vivo* doses starting from the *in vitro* actual measured concentrations provided by an *in vitro* study (Di Consiglio et al., 2020). Contrary to nominal concentrations, the actual concentrations reflect the true exposure of the cells in an *in vitro* model which is important especially after repeated exposure (Coecke et al., 2013; Kramer et al., 2015). We chose the average CPF concentration in cultured neuronal and glial cells derived from NSCs as the dose metric, since the metabolizing capacity of cultured cells at this stage of differentiation was limited and CPF intracellular concentrations did not decrease significantly with time (Di Consiglio et al., 2020).

The quantitative prediction of the *in vivo* maternal doses leading to concentrations which correspond to the *in vitro* concentration data did not match with CPF exposures measured in pregnant women with adverse developmental neurological outcomes in their children in epidemiological studies. We used data from epidemiological studies associating *in utero* CPF exposure to adverse neurodevelopmental outcomes such as learning/behavioral dysfunctions in children of exposed mothers. Since the *in vitro* non-cholinergic toxicodynamic effects were ascribed to the parent compound, CPF, we chose only studies with measured CPF blood concentrations (cord blood/mother’s blood). Studies providing a direct measurement of CPF in blood rather than the urinary excreted metabolite, TCPy could be more accurate to characterize the exposure and the dose that reaches the target tissue (Wessels et al., 2003). In this respect, few studies have measured CPF in maternal or cord blood. Silver et al. (Silver et al., 2017) described CPF blood concentrations in exposed pregnant women ranging between 0.56 and 7.33 ng/mL. In the infants, motor function was assessed at 6-week and 9-month and the scores were significantly lower for exposed *versus* unexposed infants. Chiu et al. (Chiu et al., 2021), reported an average CPF blood concentrations of 0.13 ng/mL in pregnant women and a concentration-dependent statistically significant poorer performance in the cognitive and language domains in their children at the age of 2 years in Taiwan. In the study by Rauh et al. (Rauh et al., 2006), attention deficit/hyperactivity disorder problems were observed in children and differences comparing high (above 0.011 ng/mL) *versus* low exposed children were statistically significant. Since no maternal doses (exposure levels in mg/kg bw) were described in these epidemiological studies, we applied reverse dosimetry to reconstruct the external maternal dose from the CPF blood concentrations and used it to inform the maternal-fetal model to simulate the resulting fetal brain concentrations. Accordingly, neurodevelopmental adverse effects were reported at predicted doses above 0.11 μg/kg/day of chronic exposure to CPF in those studies (considering a range of 0.00011–0.14 mg/kg bw/day oral dose). Based on BMD modelling, higher daily intake doses (i.e., 3.21–271 mg/kg bw/day) were required to induce developmental neurotoxic effects, which were captured by the *in vitro* analyzed test battery. Therefore, we conclude that the test battery used is not quantitatively predictive for developmental neurotoxic effects of CPF. The calculated dose of 20.5 mg/kg bw/day corresponding to the nominal concentration causing 5% decrease in cell viability of 21 μM, which could be considered an early event of toxicity induction was identified as the LOAEC for the analysis of synaptogenesis in the *in vitro* study (Di Consiglio et al., 2020) and is within this range. This is also higher (10–900 fold), than the LOAEL defined by the regulatory authorities, e.g., European Food Safety Authority (EFSA) based on animal studies: 0.3 mg/kg/day (EFSA, 2019). Similarly, much lower concentrations were simulated in the fetal brain (0.85–85.97 ng/mL), with an exceptional high maximum level of 1147.6 ng/mL reported in one study by Silver et al. (2017), than the IC_5_ concentration showing effects in cultured neuronal/glial cells, i.e., 21 µM (corresponding to 7942 ng/mL). It is worth noting that CPF is highly lipophilic and concentrations of CPF in blood are much lower (by orders of magnitude) than the concentration in fat or the brain tissue. In our study, the simulated brain concentrations were approximately 100 times higher than plasma concentrations based on the algorithm of Schmidt (Schmitt, 2008a; Schmitt, 2008b) implemented in the PBK modelling software.

It is also important to mention that large differences exist between *in vitro* dosing patterns and *in vivo* exposure profiles. In our study, we estimated the oral doses after multiple exposure, which is a limitation in our approach, compared to the real-world where exposure could take place simultaneously through additional routes such as inhalation and dermal routes. However, in view of the very large differences between the predicted doses and exposure levels from epidemiological studies, it is unrealistic that exposure *via* dermal and inhalation routes, from which the bioavailability of CPF is lower (e.g., dermal absorption is less than 2% of the applied dose in humans) compared to the oral route (Nolan et al., 1984; Eaton et al., 2008), could account for the high predicted doses.

For a meaningful quantitative prediction of the *in vivo* dose eliciting adverse effects *in vivo*, the *in vitro* system should adequately represent the target tissue *in vivo* as well as react with the same sensitivity. Here, the cellular model used was a culture of human iPSC-derived NSCs undergoing differentiation towards a mixed culture and neurons and glia, which is suitable to model some key processes of the developing brain (i.e., NSC proliferation, neuronal/glial cell differentiation, neurite outgrowth, synaptogenesis and neuronal network formation and function) (Bal-Price et al., 2018; Pistollato et al., 2020). Fundamental to the predictive value of an *in vitro* system is the relevance of the measured *in vitro* effects to the mode of action (MoA) of a specific chemical *in vivo*. Concerning the MoA of CPF toxicity, although AChE inhibition has been an established mechanism for toxicity, other mechanisms have been shown to play a role in neurodevelopmental adverse effects. Experimental data provide evidence that CPF can interact with and change the activity of non-AChE targets and that DNT may occur in the absence of significant AChE inhibition and even at lower concentrations (Eaton et al., 2008). For example, non-cholinesterase-depending mechanisms induced by CPF altering synaptogenesis, neuronal network formation, and BDNF signaling in differentiating PC12 cells was shown *in vitro* (at 30 μM nominal concentration) as well as in young rats *in vivo* (Betancourt et al., 2007; Slotkin et al., 2008). Indeed, CPF (at 28.5 μM) was shown to inhibit neurite outgrowth in the PC12 cells and in primary cultures of embryonic rat sympathetic neurons (Das and Barone, 1999). These data suggest that the observed effects might be considered as a reliable functional readout, specific for neuronal cell differentiation, hence, justifying its use in the *in vitro* test battery established for neurodevelopmental neurotoxicity evaluation as described in Di Consiglio et al. (Di Consiglio et al., 2020). Nominal *in vitro* CPF concentrations of 10–20 μM may lead to alterations in expression of marker genes of differentiation as shown in the study by (Estevan et al., 2013). These concentrations are in the same range as in the *in vitro* study by (Di Consiglio et al., 2020).

Our results, however, show that the predictive value of the non-cholinesterase biomarkers is low. Although they may be qualitatively related to the toxicodynamic mechanisms of CPF, they could not be utilized quantitatively to provide a meaningful PoD for risk assessment. Our work was not designed to corroborate or disprove the presumed association of CPF with neurodevelopmental impairment suggested by epidemiological studies; however, we noticed the large discrepancy between the maternal doses needed to reach the effect concentration in the fetal brain and the reported regulatory LOAEC of 0.3 mg/kg bw/day. Our fundamental aim was to evaluate the quantitative predictivity of the used *in vitro* test battery for CPF in this respect.

The potential of the *in vitro* observed effects to successfully predict *in vivo* effects relies on whether the described *in vitro* effect can be a part of the toxicity pathway. The latter is defined as biochemical pathways or molecular circuits in the cells which would lead to adverse health outcomes when sufficiently perturbed by external factors (NRC, 2007). Effects causing perturbations of the signaling pathways leading to apical endpoint alterations both qualitatively and quantitatively can be considered as key molecular mechanisms in the adverse outcome pathway (AOP) (Zhang et al., 2018).

CPF and its oxon metabolite, CPFO, are both inhibitors of brain cholinesterase activity, with CPFO being more potent (almost 1,000 times) *in vitro*. In a system of cultured sensory neurons derived from embryonic rat dorsal root ganglia, CPF was found to exert an inhibitory effect of brain AChE in concentrations of 0.1 μM (i.e., 35 ng/mL) (Yang et al., 2008). While keeping into account species-specific differences in neural/neuronal cell sensitivity to toxicants (i.e., human vs. rodent) (Baumann et al., 2016), this concentration is much lower than the concentration suggested by the non-choline esterase-toxicodynamic endpoints applied in the *in vitro* study. Nevertheless, they are in concordance with the intracellular brain concentrations in the fetus predicted from biomonitoring data where concentration as low as 0.85 ng/mL were associated with DNT. Consequently, the selected toxicodynamic markers might be less sensitive than the well-established AChE inhibition for assessment of DNT.

Another factor to consider is the contribution of CPFO, an irreversible brain AChE inhibitor, to the neurologic toxicity induced by the parent compound *in vivo*. Given the limited metabolizing capacity of brain cells *in vitro*, the role of the bioactive oxon metabolite was neglected. This could have led to overestimate the effect concentration, consequently, the doses of the parent molecule needed to induce the specific responses, which deviates significantly from the *in vivo* situation. Although the expression of some CYPs, among which CYP2B6 and CYP2C19 have been found in this *in vitro* test system (Di Consiglio et al., 2020), the content of CYPs in the brain is low compared to that in the liver (approximately 0.5%–2%) (Hedlund et al., 2001). This implies that brain CYPs have a minor overall impact on CPF kinetics *in vivo*, however, *in vitro*, this limited extent of metabolism could still be relevant for metabolites toxic to neuronal cells.

Although other CYP isoforms shown to contribute to the biotransformation of CPF into the CPFO and TCPy metabolites *in vitro*, including CYP2A6, -2C9, -2D6 and -3A4, in addition to CYP1A2 (Buratti et al., 2002; Buratti et al., 2003), they were not accounted for in our PBK model, which may be regarded as a limitation. However, the majority of isoforms (CYP2A6, -2C9, -2D6, and -3A4) were shown to be more active at high substrate concentration (Buratti et al., 2002). Therefore, they play a less significant role in metabolism at lower substrate concentrations which are characteristic of subchronic exposure rather than the acute intoxication. For instance, the oral administration of 2 mg/kg bw CPF resulted in maximum blood concentrations of about 4 ng/mL, i.e., 0.011 nM, whereas, *in vitro*, the activity of the CYP isoforms were measured at relatively higher CPF concentration ranging from 25 to 100 μM (Buratti et al., 2002) or 0.02–10 μM (Buratti et al., 2003). The underrepresentation of these isoforms might have influenced the model predictions and lead to dose underestimation in the high dose ranges. The latter, however, are not relevant in the context of repeated low dose exposure and would not reflect the *in vivo* situation.

Notably, the data yielded by epidemiological studies are subject to several limitations, which contribute to the uncertainty inherent to these studies. These limitations include the single time point analysis of the exposure biomarker, the timing of the sample collection which occurs in late-stage pregnancy/delivery rather than throughout the pregnancy, and the influence of external risk factors such as co-exposure and the social environment on the measured neurological outcomes (Reiss et al., 2015).

A further limitation to be acknowledged is the time of exposure to CPF *in vitro*. We targeted the period around 15th week of gestation to primarily mirror effects of CPF on important cellular processes in brain organogenesis taking place within this time frame captured by the *in vitro* model. This period is perceived as the window of susceptibility regarding fetal brain formation (Moore et al., 2015). It might be that prolonged *in vitro* exposure could result in lower effect concentrations than those the described in 14-day study due to cumulative effects which translates to lower *in vivo* doses. We cannot exclude CPF-induced DNT at lower doses to occur with continuous exposure. Nevertheless, given the large difference between the modelled maternal exposure doses and the resulting fetal brain concentration, on one hand and those predicted from the *in vitro* system, on the other, the impact of such limitations on our findings is less than likely.

Finally, although a human cell-relevant *in vitro* assay merits the elimination of interspecies differences, nevertheless, the differentiating cultured neuronal and glial cells derived from human NSCs might still not react with the same sensitivity to the toxic insult as the native cells *in vivo*.

In conclusion, our developed CPF PBK maternal-fetal model was capable of predicting the *in vivo* kinetics. However, the non-cholinesterase- dependent markers showed to be less sensitive than the well-known AChE inhibition in human to predict DNT in a quantitative way. Effective daily intake doses predicted from the *in vitro* model were several orders of magnitude higher than exposures estimated from epidemiological studies to induce developmental non-cholinergic neurotoxic responses analyzed in the *in vitro* test battery (Di Consiglio et al., 2020) an also higher than reported regulatory LOAEC for acetyl-cholinesterase inhibition of 0.3 mg/kg bw/day. The quantitative predictive value of the investigated non-choline esterase-dependent toxicodynamic effects, although possibly relevant for other chemicals, may not adequately represent key events in the MoA/AOPs for CPF-associated DNT.

## Data Availability

The raw data supporting the conclusion of this article will be made available by the authors, without undue reservation.
