# Supplementary material for: Prediction of in vivo prenatal chlorpyrifos exposure leading to developmental neurotoxicity in humans based on in vitro toxicity data by quantitative in vitro–in vivo extrapolation
Source: Front Pharmacol. 2023 Mar 7;14:1136174. doi: 10.3389/fphar.2023.1136174 (PMC10027916; doi:10.3389/fphar.2023.1136174)
Supplement: Supplementary file 1 [file Table1.DOCX]

***Supplementary Material***

**
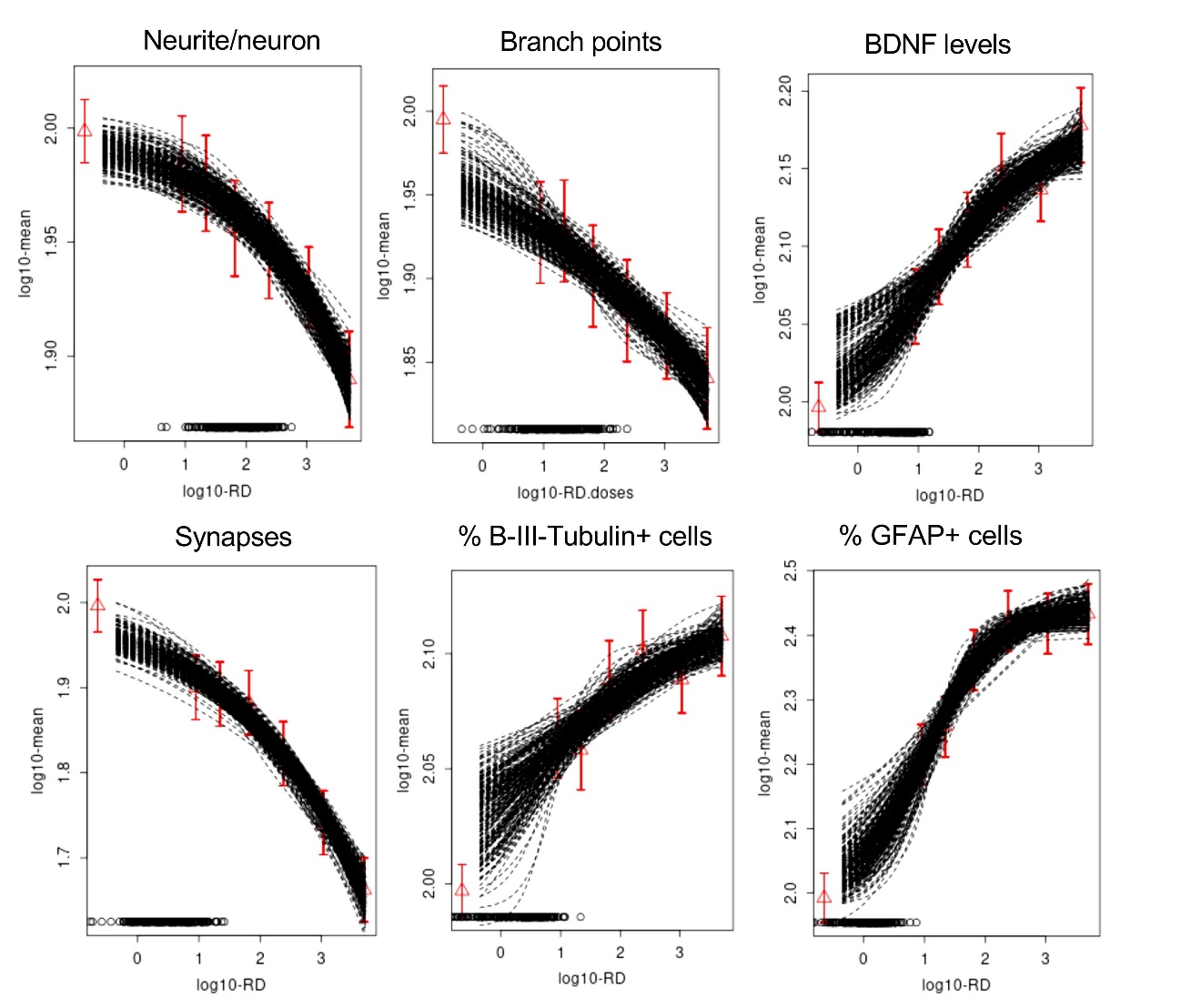
**

**Supplementary Figure 1. BMD modelling for the dose-response curves of CPF effect on the toxicodynamic biomarkers in pregnant women.** BMR= 1 SD for the respective toxicodynamic biomarker**.** Model averaging, number of bootstrap runs =200, AIC criterion 2.

**Supplementary Table 1. Computed tissue-specific partition coefficient according to the algorithm by Schmidt implemented in PK-Sim®.**

| **Organ** | **Value** |
| --- | --- |
| Bone | 67.39 |
| Brain | 307.58 |
| Fat | 2520.84 |
| Gonads | 91.77 |
| Heart | 313.50 |
| Kidney | 177.64 |
| Stomach | 200.67 |
| Small Intestine | 200.67 |
| Large Intestine | 200.67 |
| Liver Periportal | 230.22 |
| Liver Pericentral | 230.22 |
| Lung | 117.63 |
| Muscle | 39.04 |
| Pancreas | 147.29 |
| Skin | 407.12 |
| Spleen | 68.58 |
| Saliva | 0.03 |
| Myometrium | 41.42 |
| Endometrium | 154.26 |
| Breasts | 212.30 |
| PlacentaMaternal | 22.07 |
| PlacentaFetal | 22.07 |
| PlacentaMaternal | 22.07 |
| Fetus | 156.87 |

**Supplementary Table 2. Benchmark analysis for the in vitro nominal concentrations and the corresponding BMDU for the toxicodynamic endpoints.**

| **Toxicodynamic marker** | **BMC lower**  **(mg/kg bw/day)** | **BMC upper**  **(mg/kg bw/day)** |
| --- | --- | --- |
| Neurite/neuron | 21.1 | 27.4 |
| Branch points | 15.7 | 24.4 |
| BDNF levels | 10.2 | 17.1 |
| Synapses | 15.1 | 20.2 |
| % B-III-Tubulin+cells | 6.86 | 16.5 |
| % GFAP+ cells | 7.66 | 15 |

BMCU, upper limit of benchmark concentration; BMD, benchmark dose; BDNF, brain derived neurotrophic factor; GFAP, glial fibrillary acidic protein.
